# Alpha-2 adrenergic-induced changes in rectal temperature in adult and 13-day old rats following acute and repeated desipramine administration

**DOI:** 10.1186/1471-2210-8-17

**Published:** 2008-10-02

**Authors:** Jean D Deupree, William J Burke, David B Bylund

**Affiliations:** 1 Department of Pharmacology and Experimental Neuroscience, University of Nebraska Medical Center, 985800 Nebraska Medical Center, Omaha, NE 68198-5800 USA; 2 Department of Psychiatry, University of Nebraska Medical Center, 985580 Nebraska Medical Center, Omaha, NE 68198-5580 USA

## Abstract

**Background:**

The effects of acute and repeated treatment with desipramine on the functional response of α_2_-adrenoceptors were tested in adult and 13-day old rats. The functional response measured was hypothermia that was induced by brimonidine, an α_2_-adrenoceptor agonist. The change in the extent of the brimonidine-induced hypothermia following pretreatment with either single or 4 twice-daily injections of desipramine was compared in 13-day old and adult (65–75 days old) male rats.

**Results:**

Brimonidine, alone, lowered rectal temperature to a greater extent in juvenile than in adult rats, and this response was dose-dependently blocked by the selective α_2_-adrenoceptor antagonist, RX821002, in both groups of rats. Single desipramine administration lowered rectal temperature in the absence of brimonidine in adult but not in juvenile rats. The adult rats developed tolerance to this hypothermic effect after 4 days of desipramine treatment (10 mg/kg twice daily). Repeated desipramine treatment of adult rats also resulted in an enhancement in the brimonidine-induced hypothermic effect 24 h after the last dose, a time when above 90% of desipramine and its metabolite, desmethyldesipramine, had cleared the brain, but not at 14, 48 or 96 h after the last dose. In juvenile rats repeated injections of desipramine (3 mg/kg twice daily for 4 days) had no effect on the α_2_-agonist-induced hypothermia when brimonidine was given 14, 24, 63 and 96 h after the last dose of desipramine.

**Conclusion:**

The results suggest that juvenile rats response differently than adult rats to agonist stimulation of α_2_-adrenoceptors with and without pretreatment with the antidepressant desipramine. In the absence of desipramine pretreatment, the α_2_-adrenoceptor-induced hypothermic effect in juvenile rats is greater than in adult rats. Acute injections of desipramine, in the absence of agonist produced a hypothermic effect in adult but not juvenile rats. In addition, the increased α_2_-agonist-induced hypothermic effect following repeated injections of desipramine that is seen in adult rats is not seen in juvenile rats.

## Background

Although an immediate effect of tricyclic antidepressants is to inhibit the norepinephrine transporter, the antidepressant effects of the drug are most likely related to further downstream effects that occur following repeated administration. It was suggested many years ago that the effects of antidepressants are related to the down-regulation of either adrenergic and/or serotonergic receptors which occur over time suggesting that multiple steps are involved. The tricyclic antidepressant, desipramine, is known to down-regulate the β-adrenoceptor in adult rat cortices following treatment for a little as four days [1-3]. The α_1_- and α_2_-adrenoceptor densities are not affected by four days of twice daily desipramine injections in adult rats [4]. It is well known that children don't respond to the antidepressant effects of the tricyclic antidepressants [5,6]. Several studies now indicate that juvenile rats also respond differently to tricyclic antidepressants as compared to adult rats. Studies in post-natal day 13 rats demonstrate that repeated desipramine treatment down-regulates the cortical β-adrenoceptors in a manner similar to that seen in adult rats [4]; however, the α_1_-adrenoceptor in the cortex and the α_2_-adrenoceptor in the prefrontal cortex are up-regulated rather than down-regulated following 4 days of twice a day desipramine treatment [4]. Furthermore, in post-natal day 21 rats selective serotonin reuptake inhibitors, but not tricyclic antidepressants, are positive in the forced-swim test, an animal model of antidepressant action, and in the learned helplessness model of depression [7]. These results suggest that the α_2_-adrenergic systems in juvenile rats may not function in the same manner as seen in adult rats.

The purpose of this study was to determine whether the changes in α_2_-adrenoceptor densities seen following repeated desipramine injections in juvenile rats [4] result in a change in a functional response of α_2_-adrenoceptors. If so does this functional response also occur in adult rat brains, or is there a significant difference between the α_2_-adrenoceptor-mediated functional response in juvenile and adult rat brains following repeated desipramine injections. A functional α_2_-adrenoceptor response that can be easily measured in post-natal day 13 as well as adult rats is the decrease in rectal temperature. Following α_2_-agonist administration the body temperature falls several degrees over a 90 min time period and then returns to normal. Confirmation that this is an α_2_-adrenoceptor response is the fact that dexmedetomidine, an α_2_-agonist, does not cause a hypothermic response in mice lacking the α_2A_-adrenoceptor [8]. In this study the effects of acute and repeated desipramine treatment on the α_2_-agonist-induced hypothermic response was compared in post-natal day 9–12 and adult (65–75 day old) rats.

## Methods

### Drugs

Brimonidine was purchased from Sigma Aldrich (St. Louis, MO) and dissolved in DMSO and then diluted 10-fold in saline. RX821002 (Methoxyidazoxan; Tocris, Ellisville, MO) and desipramine (Sigma Aldrich, St. Louis, MO) were dissolved in water and sterilized by filtration.

### Animals

Male Sprague Dawley rats from Harlan Industries (Indianapolis, IN) were maintained on rat lab chow in conventional rat cages for 6 to 7 days prior to start of the experiment with a 12 h light dark cycle. The adult rats were between 250 g and 280 g (approximately 59–63 days old) when they arrived and were housed three to a cage, they were allowed to acclimate to the facilities for 1 week prior to use. Juvenile male rats were shipped on post-natal day 1 and arrived on post-natal day 2. The liter was culled to 9 rats on day 3. Whenever possible, 9 pups were left with the dam throughout the experiment, however, pups that were significantly underweight or that did not appear to be developing appropriately were removed. Procedures used were in strict accordance with the National Institutes of Health Guide for the Care and Use of Laboratory Animals and were approved by the UNMC Animal Care Committee.

### Hypothermic response

The adult rats (65 to 75 days old) were housed two to a wire cage during the hypothermic experiments and were prevented from lying one on top of each other. The PD13 rats were placed in an open plastic rat shipping container with divisions. Young rats maintain their body temperature by lying on top of each other or lying next to the dam. When separated from their liter mates they tend to shiver. During the hypothermic experiments the rats were not allowed to lay on top of each other because that could produce uneven changes in body temperature during the experiments. In order to help maintain a warm environment for the post-natal day 13 rats, a heating pad that controlled the environmental temperature between 28 and 30°C, was placed under the container in order to maintain a temperature comparable to what the pups experienced with the dam. The pups were allowed to move to an area that was not in contact with the heating pad so that they did not over heat as required by the animal facility. Most of the rats stayed on the heating pad although a few rats would wander around the container at times. As shown below, the heating pad did not interfere with the ability of an α_2_-adrenoceptor agonist to produce changes in rectal temperature in the juvenile rats. A heating pad was not used for the adult rats because they do not have difficulty maintaining their body temperature. The rats were grouped according to the dose of drug administered. Rats in each group were allowed to be close to each other but not on top of each other and not close to rats in other groups. If a rat moved on top of another rat, it was gently raised by the nap of the neck and rotated to the side of the other rat. For all of these experiments, rats were moved to a separate procedure room shortly before starting the experiment. All measurements of body temperature were started between 7:30 am and 8:00 am.

Rectal temperature was determined on rats that had been gently restrained by wrapping in a towel using a 0.025 inch diameter flexible probe for the juvenile rats and a 0.125 inch diameter animal rectal probe for adult rats with a Thermalert Monitoring Thermometer (Physitemp-Clifton, NJ). The equilibration time for the probes was less than a second, and the probes were held in place until the meter reading equilibrated which took less than 10 sec. An initial temperature was taken on all rats to allow them to acclimate to having their temperature taken and to the room. This initial temperature reading was not used in the data analysis. Thirty minutes later the temperature was again determined (0 time).

### Brimonidine and RX821002 administration paradigm

Except in the acute desipramine study, the decrease in rectal temperature was initiated by immediately injecting (i.p.) either brimonidine in 10% DMSO or 10% DMSO (controls). The adult rat's rectal temperature was measured 30, 60, 90 and 120 min later. The hypothermic effect lasted longer in the juvenile rats than the adult rats and therefore the temperatures for the juvenile rats were measured 30, 60, 90, and either 120 or 135 min later. Multiple doses of drugs were used in any one experiment along with two or more control rats. The number of rats used at each dose of brimonidine is given in Table 1.

**Table 1:** Administration paradigms for brimonidine and RX821002 dose-response curves.

**Age of rat at beginning of dosing**	**Drug 1**	**Dose (mg/kg)**	**Drug 2**	**Dose (mg/kg)**	**# of Rats**
	**Brimonidine**	**Dose**	**Response**		

65 to 75	Brimonidine in 10% DMSO	0	None		8
				
		0.1			6
				
		0.3			4
				
		0.6			8
				
		1.0			6
				
		1.5			7
				
		2			4

13	Brimonidine in 10% DMSO	0	None		4
				
		0.1			4
				
		0.3			4
				
		0.6			4
				
		0.8			4
				
		1.0			3

**Antagonism of**	**Brimonidine**	**by**	**RX821002**		

65 to 75	RX821002 in water	0	Brimonidine in 10% DMSO	0	8
				
		0		1	10
				
		0.02		1	4
				
		0.05		1	4
				
		0.1		1	4
				
		0.1		0	2

13	RX821002 in water	0	Brimonidine in 10% DMSO	0.6	4
				
		0.02		0.6	4
				
		0.04		0.6	4
				
		0.08		0.6	4
				
		0.08		0	4

Drug solutions were prepared so that the adult rats received 0.1 ml/100 gm and the post-natal day 13 rats received 0.1 ml/20 gm. The control rats received the same solvent as used for preparing the drug.

In some experiments, RX821002 (dissolved in water) was administered to the rats and the rectal temperature was measured at 30 min followed immediately by brimonidine administration. The rats' temperatures were then measured at the times given above. Dilutions of the drugs were prepared so the adult rats were receiving 1.0 ml/kg and the juvenile rats received 0.1 ml/20 g. The number of rats per dose for the different treatment paradigms is given in Table 1. In all experiments the change in temperature was calculated based on the temperature at 0 time.

### Desipramine administration paradigm

In the acute studies, rats' rectal temperatures were measured at 0 time, and 1 min later they were given a single injection of either water (controls) or desipramine (10 mg/kg for adult rats and 3 mg/kg for juvenile rats). The rats' temperature was measured 30 min and 60 min later. Immediately after the 60 min temperature recordings the rats were given brimonidine (1 mg/kg for adult rats and 0.6 mg/kg for juvenile rats) and the rectal temperature of the rat was measured at 30, 60, 90 and 120 min later for the adult rats and 45, 90, 135 min later for the post-natal day 13 rats. There were 4 animals in each group; however one of the post-natal day 13 rats receiving desipramine was eliminated from the analysis because of lack of a brimonidine effect (see statistical analysis section).

In the repeated desipramine treatment paradigm, rats were given an i.p. injection of either desipramine or water (controls) at about 7:30 am and 5:30 p.m. each day for 4 days (8 injections/rat). The adult rats received 10 mg/kg (10 mg/ml) of desipramine and the juvenile rats received 3 mg/kg (0.6 mg/ml) starting either the evening of post-natal day 8 or the morning of post-natal day 9. The hypothermic experiments were started at 7:30 am either 14, 24, 48, 63 or 96 hours after the last dose of desipramine. The number of rats in each group is given in the figure legends. The rats were euthanized 3 (adult rats) or 4 h (juvenile rats) after the brimonidine injection at the end of the hypothermic experiment. The brain was quickly removed and the cerebellum removed and frozen (-20°C) for later measurement of brain desipramine and the active metabolite desmethyldesipramine concentrations. The desipramine and desmethyldesipramine concentrations were measured as previously reported [9].

### Statistical methods

To determine whether the desipramine-treated animals had a different hypothermic response compared to the control animals, the change in rectal temperature over time starting at the 0 time point was calculated and the data were analyzed using a 2-way ANOVA with repeated measures using the SPSS statistical program (SPSS Inc., Chicago, IL) for changes in dose, time and time × dose. If the sphericity in the Mauchly's test was less than 0.05, the Greenhouse-Geisser test was used to determine if there was a significant difference over time, otherwise sphericity was assumed. We noticed that some animals had an abnormal response to brimonidine. Any animal who had less than 0.5 degree temperature change 90 min after the brimonidine injection was eliminated from the statistically analysis (7/81 adult rats and 1/75 juvenile rats). A few of the pups had an abnormally low weight gain over the four days. Because the brain does not develop well in underweight pups, the experimental results for any pup weighing 16 g or less on the day of the hypothermic experiment was eliminated (5/80 rats). When appropriate, a Students t-test was used to determine significant differences between drug and control conditions. To determine where there was an acute effect of desipramine in adult and juvenile rats 30 and 60 min after i.p. injection, the data were analyzed using a 2-way ANOVA (PRISM, Graphpad, San Diego, CA) followed by a Bonferroni posttest when the ANOVA results were significant.

## Results

### Dose-response relationship of brimonidine-induced decrease in rectal temperature

Initial experiments were conducted to determine the optimal dose of brimonidine for subsequent experiments. We defined the optimal dose as the dose that produced about 75% of the maximal observed hypothermic effect. The temperature of the rats was measured (0 time) just before injecting brimonidine in order to establish a baseline. Juvenile rats received doses of brimonidine between 0 and 1.0 mg/kg and adult rats between 0 to 2.0 mg/kg, and their rectal temperatures were taken at various times thereafter.

**Figure 1:**
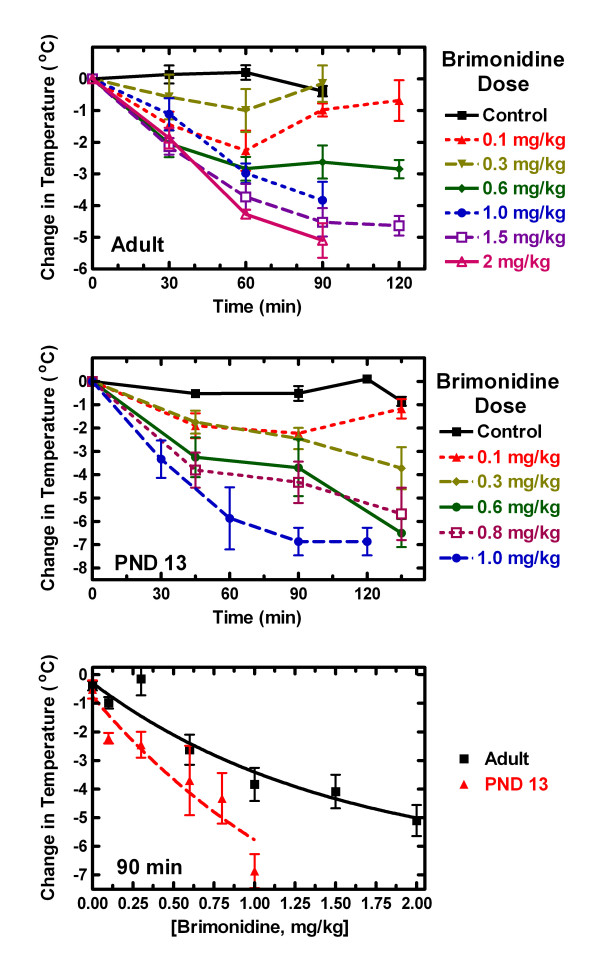
**Brimonidine-induced decrease in rectal temperature in adult and post-natal day 13 rats**. **Upper panel: **The change in rectal temperature of adult rats immediately before (0 time) brimonidine administration and 30, 60, 90 and (in some experiments) 120 min after brimonidine injection (i.p.) of 0, 0.1, 0.3, 0.6, 1, 1.5 or 2 mg/kg. The average rectal temperature for adult rats at time 0 was 35.1 ± 0.2°C and the number of rats at each dose is given in Table 1. Middle Panel:The change in rectal temperature of post-natal day 13 rats immediately before (0 time) brimonidine administration and 30, 60, 90, 120 and 135 min after brimonidine injection (i.p.) of 0, 0.1, 0.3, 0.6, 0.8 or 10 mg/kg with 3 to 6 rats per dose. The average rectal temperature at time 0 for post-natal day13 rats was 34.4 ± 0.2°C and the number of rats at each dose is given in Table 1. Lower Panel: Changes in rectal temperature in both adult and post-natal day 13 rats as a function of dose at 90 min. Data were analyzed using nonlinear regression analysis of an exponential decay equation. The two curves are statistically different using an F-test (P < 0.006). Error bars indicate S.E.M.

In adult rats, brimonidine produced a dose-dependent decrease in rectal temperature (Fig. 1). At low doses (0.1 and 0.3 mg/kg), the maximum decrease in rectal temperature occurred at 60 min with a maximum drop in rectal temperature of 2°C. By 90 min the temperature had returned to baseline. As the dose of brimonidine increased, the duration of the hypothermic effect also increased, and at the higher doses (1 to 2 mg/kg) the maximum effect occurred at 90 min. We also noted a behavioral change in the rats that received higher doses of brimonidine. At 0.3 mg/kg the rats appeared to be lightly sedated but remained standing. At 0.6 and 1.0 mg/kg the rats were sedated and were often lying down 30 min after the brimonidine injections. At 2 mg/kg the adults had shallow respiration and appeared to be asleep, yet when picked up to measure their temperature they would move around. When placed back in the cage they would become still again. The animals injected with the lower doses of brimonidine also recovered faster from the sedative effects of the drug. We did not test doses higher than 2 mg/kg because of the anesthetic-like effects of the drug. The exact EC_50 _for brimonidine cannot be calculated because of the lack of a full dose response curve. For subsequent experiments in adult rats we used a dose of brimonidine of 1 mg/kg which produces 75% of the response seen at a brimonidine dose 2 mg/kg.

The post-natal day13 rats were more sensitive to the effects of brimonidine than the adult rats as evidenced by an increased extent and longer duration of decreased rectal temperature (Fig. 1). This could be due in part to differences in rate of metabolism and/or clearance of brimonidine between the two age groups. The juvenile rats which received only 10% DMSO (control rats) maintained a relatively constant temperature (mean change = 0.4 ± 0.2°C) throughout the experiment. The maximal effect of 0.1 mg/kg brimonidine occurred at 90 min compared to 60 min in the adult rats. At higher doses (0.3 to 1 mg/kg) the temperature was still dropping at 135 min. The temperature was not measured at longer periods of time because repeated insertions of the probe were irritating to the rats. At 90 min the maximum drop in rectal temperature was 7°C at a dose of 1 mg/kg. A dose of 0.6 mg/kg brimonidine produced 74% of the 1 mg/kg effect and this dose of brimonidine was used in subsequent experiments. Behavioral effects of brimonidine in the juvenile rats could be seen at doses as low as 0.1 mg/kg. Juvenile rats receiving 0.1 mg/kg to 0.8 mg/kg initially tried to climb out of the cage but then fell asleep within 8 min of the brimonidine injection. At the lower doses of brimonidine, taking the rats temperature was enough to arouse them, and when placed back into their cage they would try to climb out again. After a few minutes they would fall back to sleep. Rats that received 1 mg/kg were sedated within 5 min of the brimonidine injection. The control (10% DMSO in saline) animals appeared to sleep normally and did not try to climb out of their cage. As with the adults, the young rats receiving the highest injection of the drug took the longest to recover. They would wake up when handled to take their temperature but then fall back to sleep when placed back in their cage.

### The hypothermic effect of brimonidine is mediated by the α_2_-adrenoceptor

**Figure 2:**
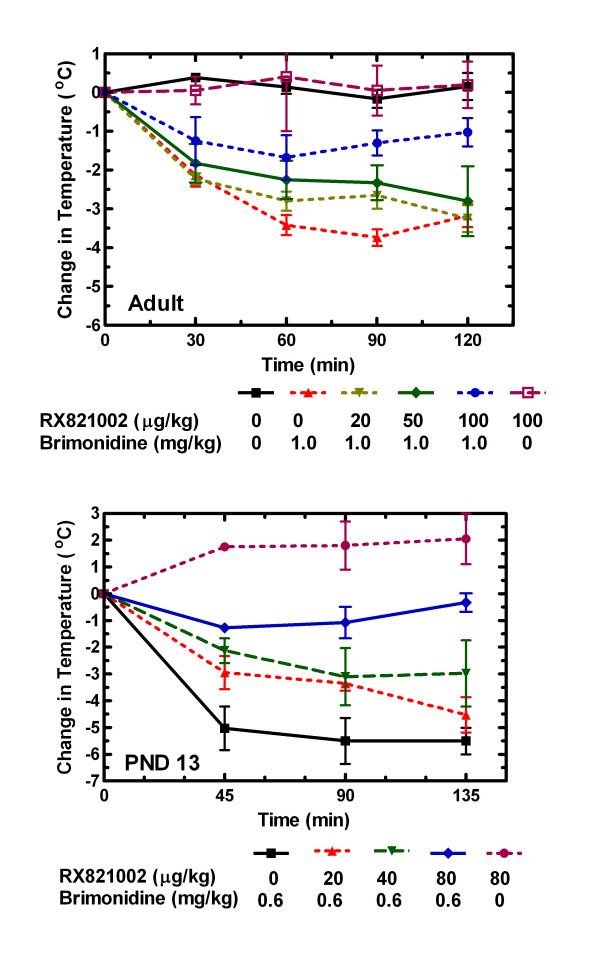
**Blockade of brimonidine effects by RX821002**. Either RX821002 or water was administered by i.p. injection 30 min prior to brimonidine. At time 0 the rectal temperature was taken, and the rat was immediately injected with brimonidine or saline. Rectal temperatures were then taken at various times thereafter, and the change in temperature from time 0 was calculated. Adult rats (upper panel; 2 to 10 rats per group). Average baseline temperature was 35 ± 0.2°C. An ANOVA of the change in temperature at the 90 min point indicates a significant difference (P < 0.001) between the 0 and the 50 and 100 μg/kg dose of RX821002 in the presence of brimonidine. However there was no statistical difference between animals receiving neither brimonidine and RX821002 and those receiving RX821002 alone (p > 0.05). Post-natal day 13 rats (lower panel). Number of rats at each dose is given in Table 1, and the average temperature of rats at 0 time was 35.0 ± 0.2°C for adult rats and 33.1 ± 0.2°C for post-natal day 13 rats. Error bars are S.E.M. An ANOVA of the change in temperature at the 90 min point indicates a significant difference (*P *< 0.01) between the 0 and the 80 μg/kg dose of RX821002 in the presence of brimonidine. However there was no statistical difference between animals receiving both brimonidine and 80 μg/kg RX821002 and those receiving RX821002 alone (*P *> 0.05).

The rats were given either water or RX821002, a selective α_2_-adrenoceptor antagonist, 30 min prior to the brimonidine injection, in order to confirm that the hypothermic effect of brimonidine is due to an action on the α_2_-adrenoceptor and not imidazoline or serotonin receptors. In this experimental paradigm rats were injected with water or RX821002 at -30 min. The rectal temperature was measured at 0 time followed by an injection of brimonidine (1 mg/kg for adult rats and 0.6 mg/kg for post-natal day13 rats). Temperatures were determined at various times thereafter. The brimonidine-induced changes from the 0 time temperature at various concentrations of RX821002 are presented in Fig. 2. In both juvenile and adult rats the extent of the hypothermic effect decreased as the dose of RX821002 increased. In adult rats, 100 μg/kg of RX821002 produced a drop in rectal temperature of 1.3 ± 0.3°C 90 min after giving brimonidine, compared to a 3.7 ± 0.2°C drop in the absence of RX821002. In the juvenile animals, a dose of 80 μg/kg of RX821002 blocked most of the hypothermic effect produced by brimonidine. In both adult and juvenile animals, RX821002 also blocked the sedative effects of brimonidine. RX821002 alone had minimal effect on the adult and post-natal day 13 rats (Fig. 2).

### Effects of acute desipramine on rectal temperature

**Figure 3:**
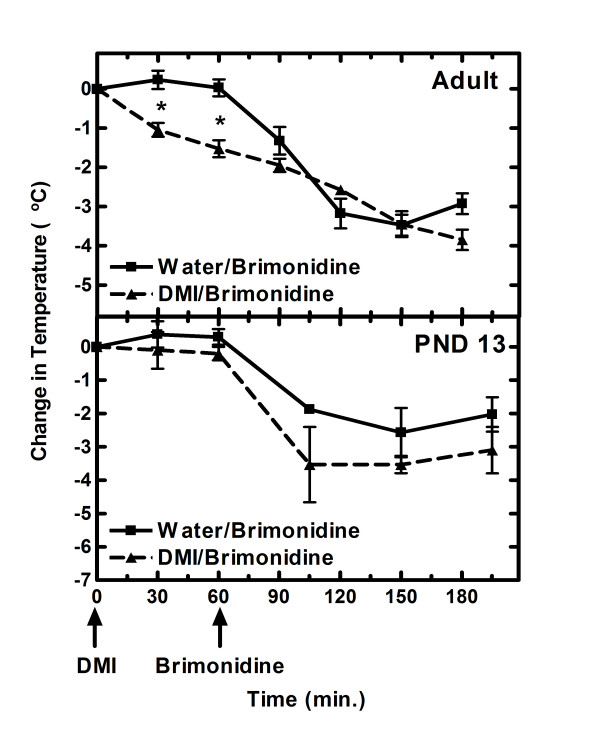
**Effect of acute desipramine on rectal temperature**. A single i.p. injection of either water or desipramine (DMI) was given to adult (10 mg/kg) and post-natal day 13 (3 mg/kg) rats immediately after measuring their rectal temperatures (0 time). The rectal temperatures were measured again at 30 min and 60 min. Immediately thereafter brimonidine was injected (1 and 0.6 mg/kg i.p. for adult and post-natal day 13 rats, respectively) and rectal temperature determined at the times noted. The mean change in rectal temperature ± S.E.M. from the zero time for each rat is given. Number of rats in the control and desipramine treated groups was 4 for adult rats and 4 and 3 for juvenile rats, respectively. *Significantly different (*P *< 0.001) than control rats at that time point and age group based on 2-way ANOVA followed by a Bonferroni post-test of the body temperatures prior to administration of brimonidine.

Because the direct action of desipramine is to block the norepinephrine transport which results in increases synaptic norepinephrine, acute administration of desipramine by itself could have hypothermic effects. Adult and post-natal day 13 rats were given a single injection of either water (control) or desipramine (10 and 3 mg/kg, respectively) immediately after determining their rectal temperatures. The rectal temperature was measured again 30 and 60 min after the desipramine injection. Previous studies have demonstrated that 3 mg/kg in post-natal day 13 rats results in similar brain desipramine concentrations to those obtained in adult rats when they are given a single acute injection of 10 mg/kg of desipramine [9]. In the adult rats there was a small, but significant drop (*P *< 0.002) in rectal temperature at 30 and 60 min (1.5°C) after the desipramine injection (Fig. 3) based on a repeated measures 2-way ANOVA followed by a Bonferroni posttest of drug effect. In juvenile animals there was no significant difference in body temperature at these two time points (Fig 3).

The presence of desipramine could also affect brimonidine-induced decreases in rectal temperature. Both the control and desipramine-treated rats were given brimonidine immediately after taking the 60 min rectal temperature and the body temperatures determined at various times thereafter. A repeat measure 2-way ANOVA of the temperature change from the time of giving desipramine until the last temperature measurement indicated no significant difference (*P *> 0.05) between drug and water treated juvenile or adult rats.

### Effect of repeated desipramine on rectal temperature

The rectal temperature immediately prior to giving brimonidine to the animals treated with desipramine for four days was compared to those of rats who received injection of water for four days (Table 2). In the adult rats the rectal temperature was 1.4°C lower (P < 0.05) 48 h after the last desipramine injection than in the control animals, however no significant differences were seen 14, 24 and 96 h after the last dose. In the juvenile animals the only significant difference (*P *< 0.05) was found 14 h after the last desipramine dose with the controls being slightly warmer (0.6°C).

**Table 2:** Comparison of desipramine-treated and control rat rectal temperatures at various times after the last dose of desipramine in the 4-day treatment paradigm.

**Adult Rats**			**Postnatal Day 13 – 16 Rats**		
**Time after last dose**	**Control**	**Desipramine**	**Time after last dose**	**Control**	**Desipramine**

**14 h**	34.4 ± 0.2 (4)	34.6 ± 0.4 (4)	**14 h**	34.2 ± 0.2 (3)	33.6 ± 0.2^a ^(5)
**24 h**	34.4 ± 0.2 (12)	34.5 ± 0.1 (12)	**24 h**	33.9 ± 0.1 (14)	33.6 ± 0.2 (12)
**48 h**	35.3 ± 0.2 (14)	33.9 ± 0.1^a ^(13)	**63 h**	35.3 ± 0.1 (11)	35.3 ± 0.3 (14)
**96 h**	34.7 ± 0.2 (8)	34.3 ± 0.1 (7)	**96 h**	35.0 ± 0.1 (13)	34.9 ± 0.2 (9)

Juvenile rats received the last injection on post-natal day 12. A student t-test was used to compare the temperatures of the control versus the desipramine treated rats prior to giving brimonidine. Values are temperature in°C ± S.E.M. Number of rats in each group is given in parenthesis.

^a^Significantly different than control at *P *< 0.05

### Effects of repeated desipramine pretreatment on brimonidine-induced decrease in rectal temperature

In order to determine whether repeated desipramine treatment altered the functional response of α_2_-adrenoceptors in juvenile and adult rats in a similar manner, both ages of rats were given desipramine or water twice daily for four days and brimonidine-induced decrease in rectal temperature was determined. The adult rats received 10 mg/kg/injection of desipramine, which is a dose previously shown by us and others to down-regulate the β-adrenoceptors [4,10] and to be effective in the learned helplessness model of depression [7]. In the post-natal day 9–12 animals, the dose of drug used (3 mg/kg/injection) has been previously shown in our laboratory to give similar brain desipramine concentration as in the adult animals receiving 10 mg/kg/injection [9]. This dose down-regulates the β-adrenoceptor, and up-regulates the α_1_- and α_2_-adrenoceptors [4]. Desmethyldesipramine, an active metabolite desipramine, is equally potent with desipramine in down-regulating the β-adrenoceptor in rats [10]. Three hours after a single acute injection of desipramine (10 mg/kg in adults and 3 mg/kg in juveniles) the concentration of desipramine was similar in both age groups (Table 3). In rats treated with desipramine for four days, 17 to 18 h after the last dose, the desipramine concentrations in the adult and juvenile rats were comparable; however the desmethyldesipramine concentration in the adult rats was five-fold higher than the juvenile rats. The clearance of the desipramine from the brain was faster in the adult animals than in the juvenile rats (Table 3) with greater than 99% elimination of the drug occurring in 52 h for the adult rats and 100 h for the juvenile rats.

**Table 3:** Concentration of desipramine and desmethyldesipramine (DDMI) in the cerebellum of rats at the end of the hypothermic experiments.

**Adult Rats**				**Post-natal Day 9–12 Rats**			
**Time ** **(h)**	**Desipramine ** **(ng/g)**	**DDMI ** **(ng/g)**	**Desipramine + DDMI ** **(ng/g)**	**Time ** **(h)**	**Desipramine ** **(ng/g)**	**DDMI ** **(ng/g)**	**Desipramine + DDMI ** **(ng/g)**

**Acute Treatment**				**Acute Treatment**			

3	1880 ± 130	430 ± 95	2310	3	1500 ± 140	100 ± 17	1600

**Repeated Treatment**				**Repeated Treatment**			

17	4010 ± 640	2710 ± 150	6720	18	3750 ± 190	530 ± 31	4280

27	220 ± 50	510 ± 79	730	28	2550 ± 480	440 ± 16	2540

52	12 ± 12	34 ± 34	46	67	82 ± 50	55 ± 18	137

100	ND	ND	ND	100	18 ± 10	2 ± 2	20

**Figure 4:**
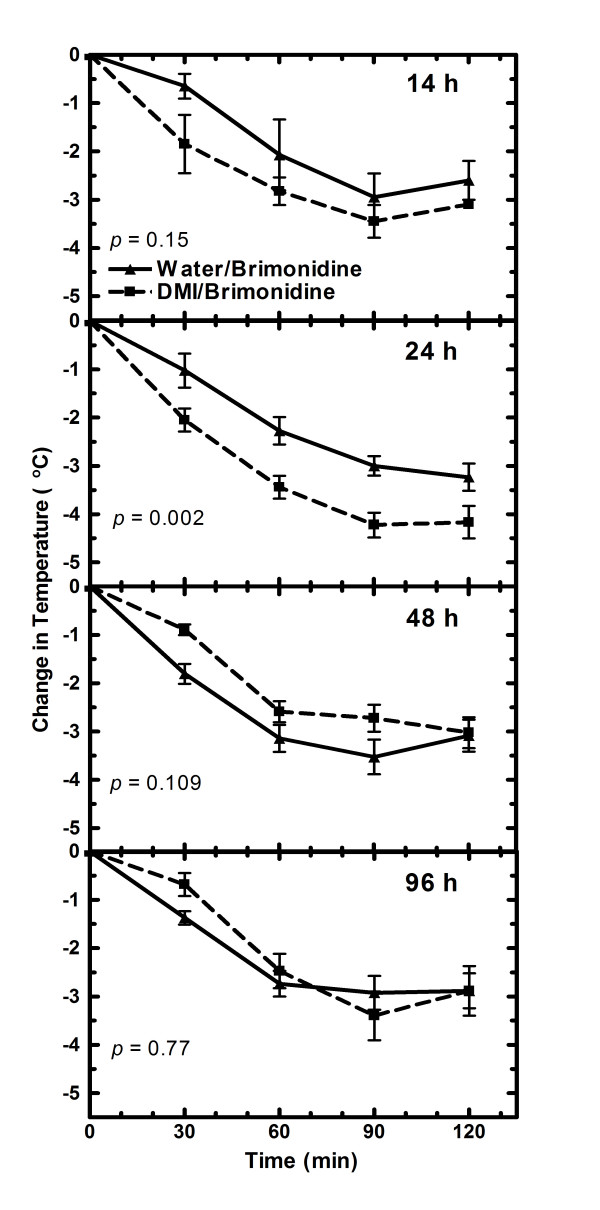
**Brimonidine induced changes in rectal temperature after 4-days of desipramine treatment in adult rats**. Rats were injected twice a day for four days with 10 mg/kg desipramine (DMI) or vehicle starting in either the morning or the evening. Measurements of rectal temperature were conducted the same time each morning 14, 24, 48 and 96 h after the last desipramine/water dose. The rectal temperature was taken immediately prior (0 time) to giving brimonidine (1.0 mg/kg) and then 30, 60, 90 and 120 min thereafter. Data points are the average change in rectal temperature from the zero time temperature for each group of rats. The number of rats used in the 14, 24, 48 and 96 h experiments were 4, 12,14 and 8 control and 4, 12, 13 and 7 desipramine treated rats, respectively. Change in temperature with drug concentration is statistically different (*P *< 0.05) 24 hr after the last dose. The change in rectal temperature with time is statistically significant (*P *< 0.001) for all experiments.

**Figure 5:**
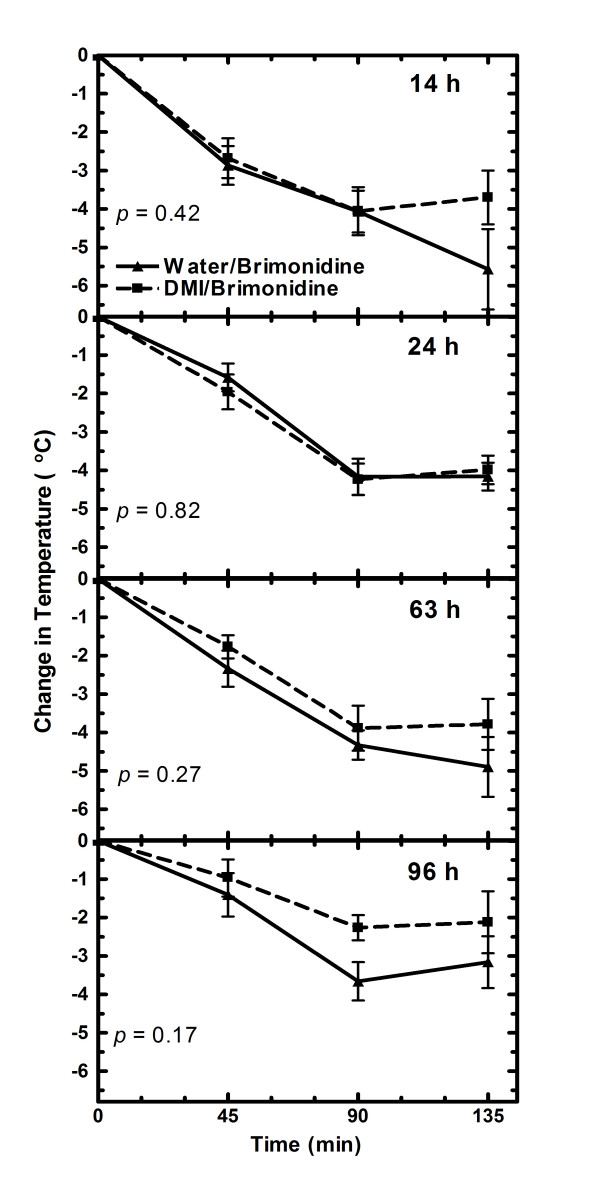
**Brimonidine induced changes in rectal temperature after 4-day desipramine treatment in juvenile rats**. Rats were injected twice a day for four days with water or 3 mg/kg desipramine (DMI) starting either the evening of post-natal day 8 or the morning of post-natal day 9. The measurements in rectal temperature were conducted the same time each morning 14, 24, 63, and 96 h after the last desipramine/water dose. The rectal temperature was taken immediately prior (0 time) to giving brimonidine (0.6 mg/kg) and then 45, 90 and 135 min thereafter. Data points are the average change in rectal temperature from the zero time temperature for each group of rats. The number of rats used in the 14, 24, 64 and 96 h studies was 3, 14, 11 and 13 control rats and 5, 12, 14 and 9 desipramine treated rats, respectively. The change in rectal temperature with time is statically significantly (*P *< 0.05) different for all experiments.

Rats that had been treated either acutely or for four days with desipramine (same animals as those in Fig. 4–5) were decapitated under isoflurane anesthesia at the completion of the hypothermic experiment (3 to 4 h after giving brimonidine) and the desipramine concentration ± S.E.M. in the cerebellum was determined. The time given in the table is the time after the last dose of desipramine. Thus the 27 hour data in this table, for example, come from the same animals as the 24 hour data in Fig. 4. The number of rats in each group is given in the legends for Fig. 1–3.

ND: Not determined, but assumed to be essentially 0.

The brimonidine-induced decreases in rectal temperature were initiated at various times after the last dose of water or desipramine because it was not known if the hypothermic effect would occur when desipramine was present in the brain and how long the hypothermic effect resulting from repeated desipramine treatment would last after desipramine and desmethyldesipramine were eliminated from the brain. In adult rats, a significantly (P = 0.002) greater hypothermic effect was obtained in treated compared to control rats 24 h after the last dose of desipramine (Fig. 4), a time when 90% of the drug and its metabolite had been eliminated from the brain (Table 3). No significant differences in the rectal temperatures were observed between control and desipramine treated rats 14 h after the last dose when the brain desipramine + desmethyldesipramine concentrations were very high or 52 h after the last dose when the desipramine + desmethyldesipramine concentrations were negligible (Fig. 4, Table 3). In contrast, in the juvenile rats there were no significant differences in rectal temperatures between rats that had received desipramine versus those only receiving vehicle at 14, 24, 63 or 96 h after the last dose (Fig. 5).

## Discussion

The most noteworthy findings of these studies are the significant differences in the α_2_-adrenoceptor induced-hypothermic response in juvenile rats as compared to adult rats after acute and repeated pretreatment with desipramine. The lack of a hypothermic response of the juvenile animals to acute desipramine treatment and the lack of effect of repeated desipramine administration on the brimonidine-induced hypothermia suggest differences in the function of the norepinephrine transporter and/or the regulation of α_2_-adrenoceptor in juvenile compared to adult rats.

### α_2_-Agonist-induced decreases in rectal temperature

In rats, a hypothermic effect can be produced by stimulating the α_2_-adrenoceptor, presynaptic 5HT_1A _[11], and possibly imidazoline receptors. Most of the hypothermic studies in rats have been conducted using compounds that are not highly selective for the α_2_-adrenoceptor, such as clonidine. Although clonidine is an α_2_-agonist, it also binds with high affinity to imidazoline sites, although this was not appreciated at the time most of the studies were conducted. The hypothermic effects of clonidine can be blocked by idazoxan [12] a compound that has a higher affinity for the imidazoline sites than the α_2_-adrenoceptor. However, the clonidine effect is also blocked by yohimbine [13], a compound with high affinity for the α_2_-adrenoceptor and low affinity for the imidazoline sites, but this compound also binds to the 5-HT_1A_, 5HT_1B_, 5-HT_1D _and dopamine D_2 _sites [14]. Mice lacking the α_2A_-adrenoceptor do not respond to the hypothermic effects of dexmedetomidine, which has both α_2_-adrenoceptor and imidazoline effects [8].

Because the purpose of this study was to look at the functional response of α_2_-agonists following repeated desipramine treatment, it was important to demonstrate the hypothermic effect was mediated by α_2_-adrenoceptors in the rat, rather than through imidazoline or serotonin mechanisms. We chose to use the brimonidine, which has good selectivity for the α_2_-adrenoceptor, although it also has some interaction with imidazoline sites. The brimonidine-induced decrease in rectal temperature was blocked by RX821002, a highly selective α_2_-antagonist that did not alter rectal temperature on its own, confirming an α_2_-adrenergic-mediated mechanism. In addition the hypothermic effect of brimonidine has been shown to be blocked by repeated infusion of antisense oligonucleotides to the α_2A_-adrenoceptor in the rat [15].

The hypothermic effects of brimonidine have not been studied previously in juvenile rats. These studies indicate that it is possible to measure a α_2_-agonists induced hypothermia in post-natal day 13 rats since the temperature of the control rats remained relatively constant throughout repeated temperature measurements and the brimonidine induced effects were significant with 4 to 7°C temperature changes at the higher doses of brimonidine. We found, that, as in adults, the hypothermic effect in juvenile rats was dose-dependent and reversed by RX821002. Major differences between the two age groups were a) a greater temperature change in the post-natal day 13 rats at lower doses of brimonidine compared to adult rats; b) the effects of brimonidine lasted longer in the post-natal day 13 rats compared to adult rats; and c) the juvenile rats exhibited behavioral effects not seen in the adult rats. It has been previously reported that clonidine produced behavioral effects in post-natal day 10 rats consisting primarily of locomotion in open areas and wall climbing [16]. These effects were blocked by phentolamine and phenoxybenzamine.

### Hypothermic effects of desipramine in juvenile compared to adult rats

Because desipramine increases synaptic norepinephrine concentrations, it would be expected to indirectly produce a hypothermic effect. Indeed, in adult rats an acute injection of desipramine produced a hypothermic effect in the absence of brimonidine. This effect of desipramine was not additive with that of brimonidine. This lack of an additive effect could be partially due to elimination of desipramine from the brain as desipramine has a half-life of elimination from the adult rat brain following a single injection of 10 mg/kg of 3.3 h [9]. Others have reported no hypothermic effect due to an acute injection of 10 mg/kg of desipramine [17-19]. In our studies, the adult rats appeared to develop tolerance to the desipramine effects with repeated injections, because rats that had twice daily injections of desipramine for four days had a similar rectal temperature to those that only received an injection of water.

Neither acute nor repeated desipramine injections produced a consistent hypothermic effect in juvenile rats, even though desipramine concentration in the juvenile brains was similar to those in the adult brains. This could be due to the fact that the norepinephrine transporter is not fully developed at post-natal day 13 [20] and thus there may have been an insufficient increase in norepinephrine to cause a hypothermic effect. Regional increases in synaptogenesis, adrenoceptor densities and norepinephrine transporter density in rats have been reported between post-natal day 10 and 20 [20]. The highest density of receptors occurs around post-natal day 20 to 30 in many regions of the brain and then declines to adult levels. Rats reach sexual maturity around post-natal day 35 which corresponds roughly to puberty or early adolescence. Another potential explanation for the lack of a desipramine effect in the juvenile rats is that α_2_-adrenoceptors are not fully functional in this age group [21]. However, this explanation is not likely because brimonidine produced a significant hypothermic effect in the post-natal day 13 rats.

### α_2_-Agonist-induced hypothermic response following repeated desipramine treatment of adult and juvenile rats

It has been previously shown that effective treatment of animals with desipramine is dependent on both dose and time of treatment [1-3]. The 4-day treatment paradigm was used in these studies because we and others have found that this paradigm causes an increase in α_1_- and α_2_-adrenoceptors in post-natal day 13 rats [4] and a decrease in β-adrenoceptor density in both adult [4,10] and juvenile animals [4]. In addition, this dose of desipramine will prevent the development of learned helplessness in adult rats [22-24]. The dose of the desipramine used in the juvenile animals was previously demonstrated to give brain concentrations of desipramine plus its active metabolite, desmethyldesipramine, similar to that found in adult rats [9]. The studies reported here confirm that 3 mg/kg/injection twice a day for four days starting on post-natal day 9 produces brain concentrations of desipramine + desmethyldesipramine at post-natal day 13 that are comparable to those seen in adult rats given 10 mg/kg/injection twice a day for four days.

It is important to note that the clearance of desipramine and desmethyldesipramine from the brain of the juvenile animals is slower than that of the adult animals. At 27 to 28 h after the last dose, the concentration of desipramine + desmethyldesipramine in the juvenile brains is 3.5 times that of the adult rats, even though the juvenile animals received a lower dose of desipramine. Over 99% of the drug is eliminated from the adult brain in 52 h, whereas it takes over 100 h to eliminate 99% of desipramine from the juvenile brains. This slow clearance of desipramine in the juvenile brain is most likely due to a lower activity of the desipramine metabolizing enzyme(s). This is further confirmed by the fact that the ratio of desmethyldesipramine/desipramine was lower in the juvenile rats compared to the adult rats suggesting that the rate limiting step is the conversion of desipramine to desmethyldesipramine and not the elimination of desmethyldesipramine.

An unexpected finding in these studies was the enhanced hypothermic effect in adult rats that was obtained 24 h after the last dose of desipramine. This effect was not seen 14 h after the last dose of desipramine when the desipramine + desmethyldesipramine brain concentrations were still high or 48 h after the last dose when the desipramine + desmethyldesipramine levels were negligible. These results suggest a transient rebound effect either on the α_2_-adrenoceptors or on down-stream mechanisms regulating body temperature. An enhanced hypothermic effect has been observed when guanfacine is used to decrease temperature after 21 days of desipramine treatment, but an attenuation of the hypothermic response when clonidine was used as the agonist [12]. Others have also reported an attenuation of the clonidine-induced hypothermic effect 24 h after repeated treatment with desipramine [12,25], imipramine [19,26] and amitripyline [27,28]. These results suggest that the effects of desipramine on drug-induced hypothermic response may depend on the agonist used, the receptors that are activated, and/or the extent of receptor activation.

The juvenile animals clearly respond differently to both the acute effects of desipramine and to the enhancement of brimonidine-induced hypothermic response after repeated desipramine treatment. In the adult animals the repeated effect of desipramine on agonist-induced hypothermic response was transient and occurred when most of the drug had been eliminated. However, in the juvenile rats, the brimonidine-induced hypothermic effect was the same in desipramine and saline-treated animals at all times studied including at 63 h when most of the drug had been eliminated.

## Conclusion

These results indicate a distinct difference in the ability of rats pretreated with desipramine to regulate the agonist induced-hypothermic response of α_2_-adrenoceptors in post-natal day 13 rats following acute and repeated desipramine treatment compared to adult rats. Potential explanations for the lack of an effect of repeated-desipramine injections in the juvenile rats are that the norepinephrine transporters and the mechanisms regulating the α_2_-adrenoceptors are not fully developed in this age group.

## Authors' contributions

JDD carried out the experiments and drafted the manuscript. WJB supervised the determination of desipramine levels and helped to draft the manuscript. DBB conceived of the study, and participated in its design and coordination and helped to draft the manuscript. All authors read and approved the final manuscript.
